# 1,213 Cases of Treatment of Facial Acne Using Indocyanine Green and Intense Pulsed Light in Asian Skin

**DOI:** 10.1155/2015/596161

**Published:** 2015-10-18

**Authors:** Kui Young Park, Ji Young Kim, Moo Yeol Hyun, Won Jong Oh, Se Yeong Jeong, Tae Young Han, Ji Young Ahn, Beom Joon Kim, Myeung Nam Kim

**Affiliations:** ^1^Departments of Dermatology, Chung-Ang University College of Medicine, 224-1 Heukseok-dong, Dongjak-ku, Seoul 156-755, Republic of Korea; ^2^Cleanup Skin Clinic, Seoul, Republic of Korea; ^3^GoodDay Skin & Laser Clinic, Seoul, Republic of Korea; ^4^Department of Dermatology, Eulji General Hospital, College of Medicine, Eulji University, Seoul, Republic of Korea; ^5^Department of Dermatology, National Medical Center, Seoul, Republic of Korea

## Abstract

*Background*. Photodynamic therapy (PDT) has been used for acne, with various combinations of photosensitizers and light sources. *Objective*. We evaluated the effectiveness and safety of indocyanine green (ICG) and intense pulsed light (IPL) in the treatment of acne. *Materials and Methods*. A total of 1,213 patients with facial acne were retrospectively reviewed. Patients received three or five treatments of ICG and IPL at two-week intervals. Clinical response to treatment was assessed by comparing pre- and posttreatment clinical photographs and patient satisfaction scores. *Results*. Marked to excellent improvement was noted in 483 of 1,213 (39.8%) patients, while minimal to moderate improvement was achieved in the remaining 730 (60.2%) patients. Patient satisfaction scores revealed that 197 (16.3%) of 1,213 patients were highly satisfied, 887 (73.1%) were somewhat satisfied, and 129 (10.6%) were unsatisfied. There were no significant side effects. *Conclusion*. These results suggest that PDT with ICG and IPL can be effectively and safely used in the treatment of acne.

## 1. Introduction

The pathogenesis of acne is multifactorial, involving follicular hyperkeratinization, increased sebum excretion, the proliferation of bacteria, and the host's innate and adaptive immunity. Acne does not occur at the site where the sebaceous glands do not exist; in other words, it is necessary to remove sebaceous glands to prevent the recurrence of acne, similar to the removal of hair roots for permanent hair removal. Therapies associated with sebum reduction are correlated with improvement in clinical acne measures. Therefore, a therapy that selectively destroys sebaceous glands to cure acne keeps developing [1]. Photodynamic therapy (PDT) is used for skin diseases. It is a medical treatment which employs the combination of light and a drug to bring about cytotoxic or modifying effects in cancerous or otherwise unwanted tissues. The reason why PDT selectively destroys target tissues without damaging neighboring tissues is due to singlet oxygen which is generated in the course of PDT. Singlet oxygen is the main treatment mechanism of PDT. Since it spreads inside a tissue, it becomes sensitive and biologically responsive only to tissues that absorb the photosensitizer. Therefore, tissues that do not contain photosensitizer do not respond to light, allowing for “selective treatment” [2]. Hongcharu and colleague suggested that topical aminolevulinic acid (ALA) PDT may inhibit sebum secretion by injuring sebaceous glands and confirmed that glands were histologically destroyed [3]. A variable combination of PDT with light sources and photosensitizers is used for acne [4].

In this study, we assessed the effectiveness and safety of PDT using indocyanine green (ICG) and intense pulsed light (IPL) for acne treatment in large number of patients.

## 2. Patients and Methods

A retrospective review was conducted on ICG and IPL treatments which were performed between January 2011 and December 2013 on a total of 1,213 patients with acne. The inclusion criteria were adult acne vulgaris patient (between 20 and 50 years old) with mild to moderate severity. Exclusion criteria were age out of range, severe acne types such as acne conglobata or fulminans, and history of any acne treatment in the preceding three months. Informed consent was obtained from all patients, and the study was approved by the hospital's medical ethics committee. The study protocol conformed to the guidelines of the 1975 Declaration of Helsinki.

The patients were asked to extract acne after cleansing and receive skin scaling for this treatment, depending on a case. A transparent wrap was used as seal in the application of 0.2% ICG cream (CU Skin, Seoul, Korea) for 15 minutes. And BroadBand Light (BBL) module of the Joule (Sciton, Inc., Palo Alto, CA) laser is illuminated right after removing the wrap. At that time, a 695 nm filter is used at 10–13 J for 30–50 msec. The treatment was given 5 times every other week. The ICG concentration is the highest in tissue after incubation and quickly eliminated as soon as topical ICG is removed so it is important to immediately investigate the light after removing the photosensitizer.

A clinical photograph was taken by the same camera at every visit. The photographs were evaluated and compared by two independent dermatologists using the quartile grading scale: 1 = minimal improvement or steady state (0–25%), 2 = moderate improvement (26–50%), 3 = marked improvement (51–75%), and 4 = excellent improvement (76–100%). Patients rated their satisfaction after treatment completion as follows: A = unsatisfied, B = somewhat satisfied, and C = highly satisfied. Any instances of adverse effects throughout the study period were recorded.

## 3. Results

**Figure 1:**
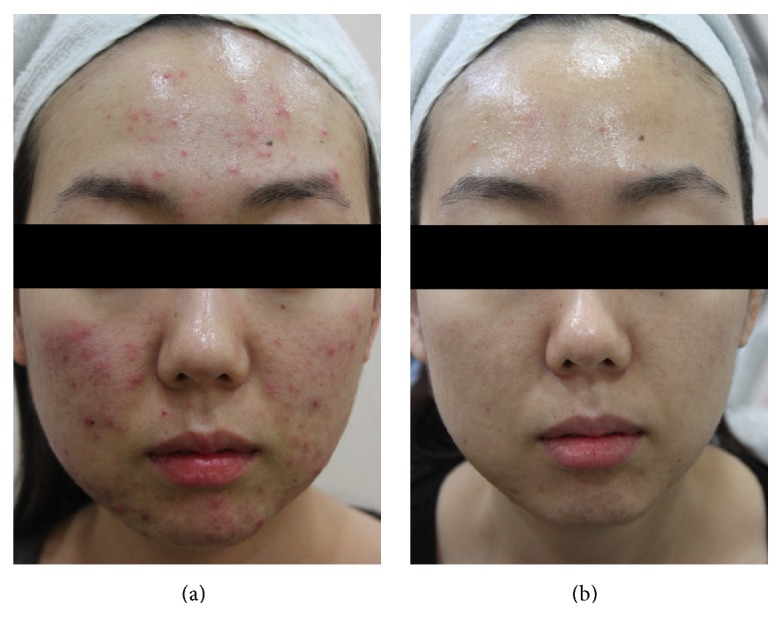
Photographs of a representative patient with acne (a) before treatment and (b) two months after final treatment.

**Figure 2:**
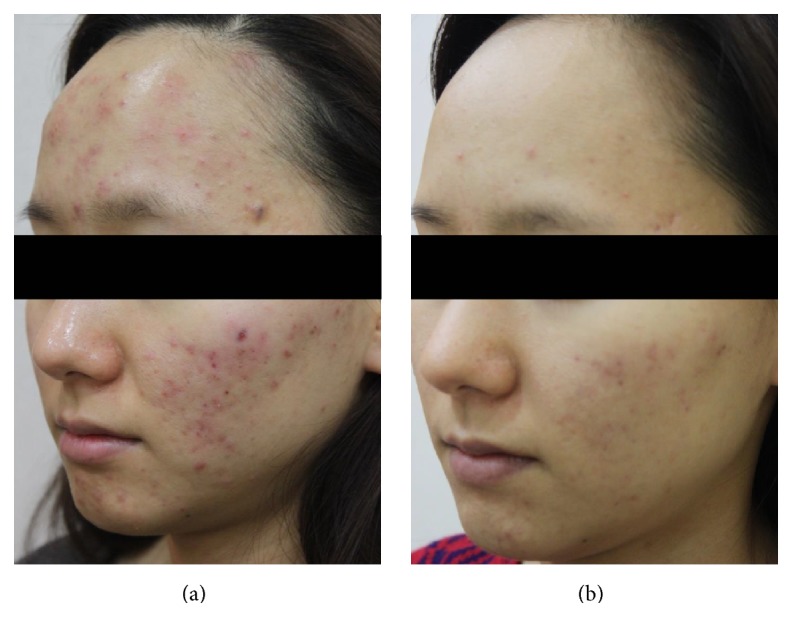
Photographs of a representative patient with acne (a) before treatment and (b) two months after final treatment.

**Figure 3:**
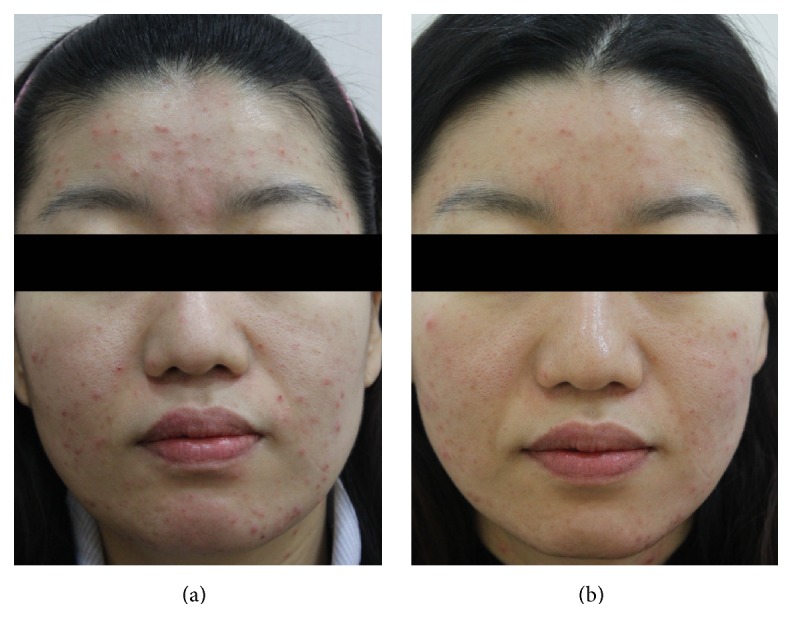
Photographs of a representative patient with acne (a) before treatment and (b) two months after final treatment.

**Figure 4:**
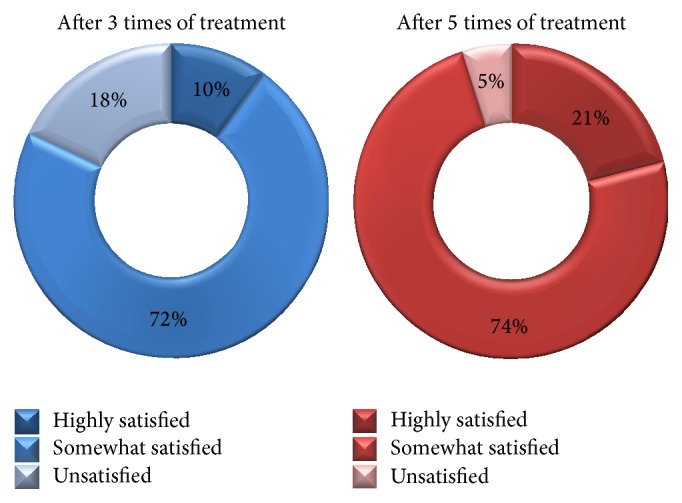
Patient satisfaction scores: 3 sessions of treatment versus 5 sessions of treatment.

A total of 1,213 subjects (934 females and 279 males) aged 13–44 years (mean age of 25.7 years) of skin types III–V with acne were enrolled. The two independent dermatologists' comparisons of the baseline and posttreatment photographs showed that there was more than minimal improvement in all of the patients. Marked to excellent improvement was noted in 483 of 1,213 (39.8%) patients (Figures 1 and 2), while minimal to moderate improvement was achieved in the remaining 730 (60.2%) patients (Figure 3). None of the patients reported a lack of change or a worsening of their acne. The mean improvement score was 2.4. Patient satisfaction scores revealed that 197 (16.3%) of 1,213 patients were highly satisfied, 887 (73.1%) were somewhat satisfied, and 129 (10.6%) were unsatisfied. More patients responded “highly satisfied” after 5 sessions of treatment than after 3 sessions of it (Figure 4). The treatments were well tolerated with only minor side effects during and after the treatments including pain, erythema, scales, and pruritus. These resolved within a week without any treatment.

## 4. Discussion

The pathogenesis of acne is multifactorial, and among the causative factors, the sebaceous gland plays an important role in the initiation of the disease, as it possesses all of the enzyme machinery for the production of hormones and cytokines. Most of the current clinical therapies for acne actively use heat to destroy sebum. Sebaceous glands residing in the upper dermis are believed to be heat-sensitive; that is, heating sebaceous glands with a long wavelength 1450 nm diode laser causes them to shrink and produce less sebum. In addition, thermal coagulation of the sebaceous lobule by 1450 nm diode laser reduces sebaceous gland activity that subsequently leads to a reduction in inflammatory acne lesions. When a fine insulation needle of high frequency is used to produce selective electrothermolysis, sebum secretion can be eliminated and acne can be cured [5, 6]. Dilated vessels and oxyhemoglobin are also targets for red light, which results in injury to the sebaceous glands at the sites of acne lesions [7]. Although the mechanism is not completely understood, the efficacy of PDT in the treatment of acne has been established. Also, clinical data have been correlated with amount of sebum excretion and degree of sebaceous glands' damage [8, 9].

Therapeutic window in PDT is also called the optic window which encompasses the range of wavelengths from 600 to 1300 nm. Primarily due to the lack of strong absorption by blood and secondarily due to lower optical scattering within the dermis, the absorption coefficient of tissue is determined by the concentration of light-absorbing molecules (chromophores). At PDT wavelengths, the two most important chromophores are hemoglobin and water, although lipids, melanin, and other pigments such as bilirubin can also make significant contributions in some cases. The collateral heating may cause pain and other undesirable consequences. A lack of indigenous chromophore at this range of wavelengths implies that the target has to be selectively loaded prior to laser treatment with appropriate exogenous chromophore. The current clinical trend is to develop therapeutic windows that are less absorptive of water, hemoglobin, and melanin, namely, near-infrared absorbing photosensitizer, and to cure acne tissues with a light source of longer wavelength [9, 10].

In general, the “ideal” PDT agent has some properties. First thing is high absorptivity at long wavelengths which have range from 700 to 850 nm for maximum light penetration in tissue. ICG, a nontoxic tricarbocyanine dye, meets this criterion and is considered to be a very safe chromophore due to its low incidence of adverse effects. It has been applied in many fields such as measurement of cardiac output, angiography, and photocoagulation. ICG is also easily absorbed, quickly distributed throughout the treated tissues, and excreted from the body. Abels et al. reported that the activation of ICG by a near-infrared diode laser was due to photodynamic action meaning that it generates free oxygen radicals which result in photooxidation [11]. Also, Fickweiler et al. demonstrated that the photodynamic action of ICG effectively kills HaCaT keratinocytes through the generation of reactive oxygen species, especially singlet oxygen molecules. In other words, ICG is able to produce powerful photosensitized cellular damage [12].

Topically applied ICG is highly absorbed and accumulated in the sebaceous glands through the follicular pores. Penetration time of the dye is 5–15 min at a depth of 1 mm. The use of a laser with ICG for treatment of acne has been reported by three groups. Lloyd and Mirkov evaluated the effect of long-pulse 810 nm diode laser on the enlarged sebaceous glands of a single patient preloaded with ICG [13]. After confirming the penetration of ICG into the enlarged gland, the authors performed a laser-tissue interaction analysis to determine the appropriate treatment parameters to selectively damage the enlarged ICG-loaded glands. Fluorescence microscopy and histological studies of the treated areas revealed selective necrosis of the targeted glands, whereas clinical observations and serial photographs showed an improvement in acne after the treatment. The authors concluded that the diode laser had selectively and safely damaged the enlarged sebaceous glands. Tuchin and colleagues reported that multiple treatment sessions yielded more favorable results than a single session and attributed the efficacy to an antibacterial effect [14]. In a similar study, Genina and colleagues reported that the ICG-diode laser protocol provided the best results in patients with moderate to severe acne [15]. The consistent results of these studies support the conclusion that the ICG-diode laser is a valid option for the treatment of moderate to severe acne.

In fact, the effectiveness of ALA PDT using IPL for treatment of moderate to severe inflammatory acne was reported in 2004 [16]. Since then, not only blue light with peak absorption by the photosensitizer but also those lights with smaller peak absorption have been widely used for PDT. Hypothetically, sufficient light energy delivered into any portion of the absorption band should activate or initiate photodynamic effects. IPL has emission spectra of 400–1400 nm, depending on the filter. Particularly wavelengths in the red and near-infrared spectrum might be suitable for excitation of ICG whereas ICG has rather little absorption at wavelengths below 600 nm. ICG light absorption is maximal at approximately 800 nm and decreases for shorter wavelengths. But there is still sufficient light absorption in the ICG molecule especially from 600 to 700 nm. Therefore, once a photon is absorbed by ICG, singlet oxygen can be generated and thus activate the ICG molecule [17].

Recently, many patients in our clinic seek the least invasive and fastest treatments for improving inflammatory acne and its remnants. Since IPL has fast efficacy and high satisfaction of patients because of its uniformed illumination and rejuvenation, we started to use BBL's near-infrared filter to activate ICG. By considering the absorption curve of ICG, the depth of BBL's cut-off filter, and the location of sebaceous glands where ICG is embedded, the cut-off filter of 695 nm was chosen for the treatment. There is one study that examined the efficacy of ICG PDT with an IPL on capillary malformation. In the study, only one patient showed poor to moderate clearance and slightly improved cosmetic appearance after IPL therapy alone and IPL + ICG treatment. With regard to IPL therapy, four patients rated IPL therapy alone and IPL + ICG as equally effective or ineffective; thus IPL + ICG therapy had no beneficial effect when compared to IPL treatment alone [18]. This result is assumed to be due to the fact that the study's treatment aimed to activate ICG in the vessels after systemic venous injection, rather than for topical PDT. Therefore, the study used an IPL of 555–950 nm. The emitted wavelength band of IPL was suitable for absorption in hemoglobin and ICG, but this dual absorption blocked ICG from activating, playing a role analogous to sunscreen. In other words, ICG and hemoglobin act as competitive chromophores with each other in this case.

The lightening of skin observed after the first session is the greatest merit of using ICG PDT with IPL and is also the greatest point of difference from diode for light source. According to our survey, bumps, pus, and postinflammatory hyperpigmentation were characteristics ranked most troublesome in adult female acne. Given IPL's whitening effects (i.e., a lightening of basic skin tone), IPL treatments are thought to result in more subjective satisfaction in acne patient. However, it remains unclear whether this whitening effect is due to PDT with ICG or IPL alone. Despite the well-known effectiveness of laser-based methods, particularly for Asians as compared to Caucasians, the debate continues on the relative advantages of IPL treatments for acne and on the modes of action involved in their clinical efficacy. IPL with or without photosensitizer has also been recommended as a safe and effective modality for the treatment of acne, especially in Caucasians, although studies of its efficacy on Asian skin have shown no significant improvements. This discrepancy may result from Asian skin having a different response to light than Caucasian skin or the use of inappropriate wavelengths or inaccurate targeting of the light source. Chang and colleagues reported that IPL alone was effective for acne red macules, pigmentation, and skin tone but did not affect inflammatory acne lesion counts on the skin of Korean patients [19].

Double treatment with PDT is also possible, with short and long pulses and appropriate adjustments in the concentration of the photosensitizer. Depending on the situation, a stronger treatment can be applied with a high concentration of photosensitizer by intralesional injection or additional treatment for the severe area selectively with a diode laser after using the IPL on the whole face generally. Despite the fact that the effective output power is more reduced at the photosensitizer activation peak compared with a diode laser source, ICG-IPL is a good option for the control of acne due to its fast clinical efficacy, very high satisfaction scores among patients, and lack of side effects. Further work on the treatment of acne using ICG and IPL is needed to determine the proper parameters as well as find histological evidence of efficacy and ways to effectively deliver ICG. In addition controlled clinical trials will be required in the future.

## References

[1] Makrantonaki E., Ganceviciene R., Zouboulis C. (2011). An update on the role of the sebaceous gland in the pathogenesis of acne. *Dermato-Endocrinology*.

[2] Choudhary S., Nouri K., Elsaie M. L. (2009). Photodynamic therapy in dermatology: a review. *Lasers in Medical Science*.

[3] Hongcharu W., Taylor C. R., Chang Y., Aghassi D., Suthamjariya K., Anderson R. R. (2000). Topical ALA-photodynamic therapy for the treatment of acne vulgaris. *Journal of Investigative Dermatology*.

[4] Riddle C. C., Terrell S. N., Menser M. B., Aires D. J., Schweiger E. S. (2009). A review of photodynamic therapy (PDT) for the treatment of acne vulgaris. *Journal of Drugs in Dermatology*.

[5] Laubach H.-J., Astner S., Watanabe K. (2009). Effects of a 1,450 nm diode laser on facial sebum excretion. *Lasers in Surgery and Medicine*.

[6] Yeung C. K., Shek S. Y., Yu C. S., Kono T., Chan H. H. (2009). Treatment of inflammatory facial acne with 1,450-nm diode laser in type IV to V Asian skin using an optimal combination of laser parameters. *Dermatologic Surgery*.

[7] Na J. I., Suh D. H. (2007). Red light phototherapy alone is effective for acne vulgaris: randomized, single-blinded clinical trial. *Dermatologic Surgery*.

[8] Taub A. F. (2007). Procedural treatments for acne vulgaris. *Dermatologic Surgery*.

[9] Pollock B., Turner D., Stringer M. R. (2004). Topical aminolaevulinic acid-photodynamic therapy for the treatment of acne vulgaris: a study of clinical efficacy and mechanism of action. *British Journal of Dermatology*.

[10] Taylor M. N., Gonzalez M. L. (2009). The practicalities of photodynamic therapy in acne vulgaris. *British Journal of Dermatology*.

[11] Abels C., Fickweiler S., Weiderer P. (2000). Indocyanine green (ICG) and laser irradiation induce photooxidation. *Archives of Dermatological Research*.

[12] Fickweiler S., Szeimies R.-M., Bäumler W. (1997). Indocyanine green: intracellular uptake and phototherapeutic effects in vitro. *Journal of Photochemistry and Photobiology B: Biology*.

[13] Lloyd J. R., Mirkov M. (2002). Selective photothermolysis of the sebaceous glands for acne treatment. *Lasers in Surgery and Medicine*.

[14] Tuchin V. V., Genina E. A., Bashkatov A. N., Simonenko G. V., Odoevskaya O. D., Altshuler G. B. (2003). A pilot study of ICG laser therapy of *acne vulgaris*: photodynamic and photothermolysis treatment. *Lasers in Surgery and Medicine*.

[15] Genina E. A., Bashkatov A. N., Simonenko G. V., Odoevskaya O. D., Tuchin V. V., Altshuler G. B. (2004). Low-intensity indocyanine-green laser phototherapy of acne vulgaris: pilot study. *Journal of Biomedical Optics*.

[16] Gold M. H., Bradshaw V. L., Boring M. M., Bridges T. M., Biron J. A., Carter L. N. (2004). The use of a novel intense pulsed light and heat source and ALA-PDT in the treatment of moderate to severe inflammatory acne vulgaris. *Journal of Drugs in Dermatology*.

[17] Engel E., Schraml R., Maisch T. (2008). Light-induced decomposition of indocyanine green. *Investigative Ophthalmology and Visual Science*.

[18] Klein A., Bäumler W., Buschmann M., Landthaler M., Babilas P. (2013). A randomized controlled trial to optimize indocyanine green-augmented diode laser therapy of capillary malformations. *Lasers in Surgery and Medicine*.

[19] Chang S.-E., Ahn S.-J., Rhee D.-Y. (2007). Treatment of facial acne papules and pustules in Korean patients using an intense pulsed light device equipped with a 530- to 750-nm filter. *Dermatologic Surgery*.

